# Postnatal Loss of Neuronal and Glial Neurofascins Differentially Affects Node of Ranvier Maintenance and Myelinated Axon Function

**DOI:** 10.3389/fncel.2017.00011

**Published:** 2017-02-03

**Authors:** Anna M. Taylor, Julia Saifetiarova, Manzoor A. Bhat

**Affiliations:** Department of Cellular and Integrative Physiology, Center for Biomedical Neuroscience, School of Medicine, University of Texas Health Science CenterSan Antonio, TX, USA

**Keywords:** myelin, axonal degeneration, nodes, paranodes, neurofascin

## Abstract

Intricate molecular interactions between neurons and glial cells underlie the creation of unique domains that are essential for saltatory conduction of action potentials by myelinated axons. Previously, the cell surface adhesion molecule Neurofascin (Nfasc) has been shown to have a dual-role in the establishment of axonal domains from both the glial and neuronal interface. While the neuron-specific isoform of Neurofascin (NF186) is indispensable for clustering of voltage-gated sodium channels at nodes of Ranvier; the glial-specific isoform of Neurofascin (NF155) is required for myelinating glial cells to organize the paranodal domain. Although many studies have addressed the individual roles of NF155 and NF186 in assembling paranodes and nodes, respectively; critical questions about their roles in the maintenance and long-term health of the myelinated axons remain, which we aimed to address in these studies. Here using spatiotemporal ablation of Neurofascin in neurons alone or together with myelinating glia, we report that loss of NF186 individually from postnatal mice leads to progressive nodal destabilization and axonal degeneration. While individual ablation of paranodal NF155 does not disrupt nodes of Ranvier; loss of NF186 combined with NF155 causes more accelerated nodal destabilization than loss of NF186 alone, providing strong evidence regarding a supporting role for paranodes in nodal maintenance. In both cases of NF186 loss, myelinating axons show ultrastructural changes and degeneration. Our studies reveal that long-term maintenance of nodes and ultimately the health of axons is correlated with the stability of NF186 within the nodal complex and the presence of auxiliary paranodes.

## Introduction

The clustering of proteins into distinct molecular domains along myelinated axons allows for the segregation of voltage-gated ion channels, which is critical for saltatory conduction. The molecular components of axonal domains, including axon initial segments (AIS), nodes of Ranvier (nodes), paranodes, and juxtaparanodes, have been well established (Lambert et al., 1997; Rasband et al., 1998; Pedraza et al., 2001; Thaxton and Bhat, 2009). The key components at nodes are the 186 kDa neuron-specific isoform of Neurofascin (*Nfasc*^*NF186*^), Ankyrin-G (AnkG), βIV Spectrin and voltage-gated sodium (Na_V_) channels. While at the paranode, Contactin, Contactin-associated protein (Caspr), and the 155 kDa glia-specific isoform of Neurofascin (*Nfasc*^*NF155*^) are critical for maintaining the interface between the axon and myelinating glial cells.

To precisely ascertain how molecular domains are organized, mouse models lacking these individual components have been generated (reviewed in Buttermore et al., 2013). Mice deficient for the cell adhesion molecule Neurofascin (*Nfasc*), which lack both NF155 and NF186 isoforms, were found to die around postnatal day 7 (P7) when the transition to saltatory conduction occurs (Sherman et al., 2005). Despite being unable to establish nodes or paranodes, *Nfasc* knockouts have normal levels of myelination (Zonta et al., 2008). In order to address the individual roles of NF155 and NF186, we generated *Nfasc*^*Flox*^ mice (Pillai et al., 2009). Neuron-specific Cre-dependent ablation of NF186 during development led to lack of Na_V_ channels and AnkG at nodes, paranodal invasion into the nodes, a significant reduction in nerve conduction velocity (NCV), and death around P20 (Thaxton et al., 2011). Loss of NF155 through a myelinating glia-specific Cre resulted in mice that lack paranodes and are unable to segregate nodal Na_V_ channels from juxtaparanodal voltage gated potassium (K_V_) channels. Although these mice had fully developed nodes, they displayed loss of axo-glial junctions, severe reductions in NCV, and died around P17 (Pillai et al., 2009; Thaxton et al., 2011). Together these results provide strong evidence for a pioneering role for NF186 at the node and NF155 at the paranode.

Although many studies have addressed the initial axonal domain assembly during development, investigations into the long-term maintenance of axonal domains throughout life have just begun. Using inducible myelinating glia-specific Cre, NF155 postnatal ablation led to a gradual degradation of NF155 and progressive disruption of the paranodal axo-glial junctions. This also caused loss of segregation of juxtaparanodal K_V_ channels from the nodal Na_V_ channels and a modest but significant reduction in NCV (Pillai et al., 2009). Recently utilizing an adult neuronal ablation model of NF186, Desmazieres et al. (2014) reported that loss of NF186 did not lead to a total absence of Na_V_ channels at nodes of Ranvier in either the central nervous system (CNS) or peripheral nervous system (PNS) suggesting the nodal protein complex could be maintained without the extended presence of NF186. Despite the persistence of nodal proteins, significant reduction in conduction velocity, as well as, death of the mice 4 months after ablation was reported. Additionally, paranodes were suggested to play a role in nodal maintenance (Desmazieres et al., 2014); however, the consequences of adult ablation of a paranodal component along with NF186 remain to be addressed. Overall these studies indicate that NF155 and NF186 contribute to the stability of axonal domains in adults; however, whether long-term loss of Neurofascin leads to axonal degeneration as is seen in demyelinating disorders remains unclear.

In the current study to address the relative contributions of NF186 and the paranodal axo-glial junctions toward the life-long stability of the nodal complex as well as the consequences of adult nodal disorganization on the health of the myelinated axons, we utilized neuronal-specific, myelinating glia-specific, and ubiquitous inducible Cre lines. With the neuronal-specific line, we demonstrate that ablation of NF186 from mature nodes leads to progressive nodal disorganization and extensive axonal degeneration, along with loss of NCV and premature death around 6 months post injection (mpi). The consequences of nodal deterioration are further accelerated, when NF186 and NF155 are simultaneously ablated after postnatal development; however, no nodal destabilization is observed after NF155 ablation even 12 mpi. Our results provide evidence that NF186 remains remarkably stable at the nodes for months after ablation with intact paranodes, but when NF186 is eventually lost, this induces rapid nodal destabilization and neurodegeneration. Together, our findings suggest that the stability of the nodal complex is dependent on NF186 and that the stability of NF186 is enhanced when the flanking paranodal domains are present. These data provide insights into life-long nodal maintenance and axonal health, and how progressive demyelinating conditions compromise axonal domains leading to degeneration of myelinated axons and onset of neurological pathologies.

## Materials and methods

### Materials

Unless otherwise noted, all chemicals and reagents were purchased from Sigma Aldrich (St. Louis, MO). Details for all antibodies used in this work are listed in Table 1.

**Table 1:** **List of Antibodies used in the current studies**.

**Antibody (Concentration)**	**Immunogen**	**Manufacturer**
anti-α-Tubulin (1:10,000 WB)	full length α-Tubulin (tetrahymena)	DSHB # 12G10 (Mouse IgG_1_, monoclonal)
anti-Ankyrin G (1:500 IMF; 1:1500 WB)	amino acids TEDK-KKTH	unpublished (Rabbit, polyclonal)
anti-Ankyrin R (1:200 IMF)	full length AnkR (human)	NeuroMab #75-380 (Mouse IgG_2b_, monoclonal)
anti-βIV Spectrin (1:1000 IMF; 1:1000 WB)	amino acids ARRA-QESA	unpublished (Rabbit, polyclonal)
anti-Caspr1 (1:500 IMF)	cytoplasmic domain (mouse)	Bhat et al., 2001 (Rabbit and Guinea Pig, polyclonal)
anti-Caspr1 (1:100 IMF)	amino acids QNHR-SRSE, cytoplasmic domain	NeuroMab #75-001 (Mouse IgG_1_, monoclonal)
anti-K_V_1.2 (1:200 IMF)	amino acids QYLQ-LTDV, cytoplasmic domain	NeuroMab #75-008 (Mouse IgG_2b_, monoclonal)
anti-Myelin Basic Protein (1:1500 WB)	cytoplasmic domain (mouse)	Abcam #ab40390 (Rabbit, polyclonal)
anti-Na_V_ channels pan (1:500 IMF; 1:1500 WB)	amino acids FNQQ-AFDI of Na_V_1.6	unpublished (Rabbit, polyclonal)
anti-Neurofascin-186 (1:500 IMF)	amino acids TVGT-VYSR, mucin domain	Thaxton et al., 2011 (Guinea Pig, polyclonal)
anti-Neurofascin pan; NFCT (1:500 IMF; 1:2,000 WB)	amino acids FIKR-YSLA, cytoplasmic domain	Pillai et al., 2009 (Rat and Guinea Pig, polyclonal)

*IMF, Immunofluorescence; WB, Western Blot*.

Briefly, polyclonal antibodies were generated to AnkG from amino acids TEDK to KKTH, to βIV Spectrin from amino acids ARRA to QESA, and to pan-Na_V_ channels from amino acids FNQQ to AFDI of the sodium channel Na_V_1.6. Other antibodies used are anti-Caspr (Bhat et al., 2001), anti-NF186 (Thaxton et al., 2011), and anti-NFCT (Pillai et al., 2009). Commercial antibodies used included rabbit anti-myelin basic protein (Abcam; Cambridge, MA); mouse anti-α-tubulin (DSHB; Iowa City, IA); mouse anti-Caspr, anti-AnkR and anti-Kv1.2 (NeuroMab; Davis, CA), fluorescent secondary antibodies (Alexa Fluor; Life Technologies; Grand Island, NY), and infrared (IR) conjugated secondary antibodies (LI-COR; Lincoln, NE). All electron microscopy reagents were purchased from Electron Microscopy Sciences (Hatfield, PA).

### Animals and treatments

To characterize the role of Neurofascin in nodal maintenance, *Nfasc*^*Flox*/−^ (*NF*^*fx*/−^) mice (Pillai et al., 2009) were bred to three inducible Cre lines with specific patterns of expression: *Plp-CreER* [expressed in only myelinating glial cells; (Doerflinger et al., 2003)], *SLICK-H-CreER* [a derivative of *Thy1.2-CreER* expressed in projection neurons; (Heimer-McGinn and Young, 2011)], and ubiquitously expressed *Actin-CreER* (Hayashi and McMahon, 2002). Mice were group-housed in the animal facility with temperature-controlled rooms (23 ± 1°C) and a maintained light cycle (12 h light on/12 h off). The mice were allowed *ad libitum* access to water and a standard rodent diet. Mice were maintained on a mixed strain background of C57BL/6 and 129/Sv, and genotyped before weaning at 21 days of age. To induce genetic ablation of NF155, NF186, or both, 1 mg tamoxifen (MP Biomedicals) suspended in sunflower seed oil was delivered as intraperitoneal (ip) injections for 10 consecutive days between P23–32. At various timepoints post-injection, these mice were evaluated by electrophysiological, immunohistochemical, biochemical, and ultrastructural techniques. Aged matched non-Cre *NF*^*fx*/−^ littermates (who also received tamoxifen injections) were used as controls. These studies utilized equal numbers of males and females per group unless noted otherwise. All mice were provided with moist food on the floor of their cage to promote survival. End stage was defined as the point at which mice placed on their backs could no longer right themselves. At this point, mice were humanely euthanized. All animal research was conducted in conformity with the Public Health Service Policy on Humane Care and Use of Laboratory Animals, and experiments were performed with prior approval from the Institutional Animal Care and Use Committee of the University of Texas Health Science Center at San Antonio (protocol #12092x).

### Immunofluorescence

At various timepoints post-tamoxifen, *CreER;NF*^*fx*/−^ and controls were anesthetized through an intraperitoneal injection of Avertin (2-2-2 tribromoethanol in 2-methyl-2-butanol). Once mice no longer responded to touch, the right sciatic nerve (SN) was removed and placed in 4% paraformaldehyde (PFA) made in 0.01M phosphate buffer saline (PBS). Mice were perfused intracardially using a peristaltic pump for 3 min with saline followed by 2 min with a chilled 0.1M phosphate buffer (PB) containing 1% PFA and 1% sucrose.

#### Sciatic nerve

After 30 min in 4% PFA on ice, the SN was washed at least three times with PBS. The individual nerve fibers were teased apart on slides and allowed to dry overnight. The teased nerves were then placed in a humidity chamber at room temperature and incubated for 1 h in blocking buffer (5% bovine serum albumin, 1% goat serum, and 0.2% triton x-100) before overnight incubation with primary antibodies for nodal, paranodal, or juxtaparanodal proteins diluted in blocking buffer. After three PBS washes, nerves were incubated in the dark with corresponding Alexa Fluor secondary antibodies at 1:1000 for 2 h. Following 3 more washes, slides were coverslipped using anti-fade mounting media (0.2% n-propyl gallate, PBS, and 90% glycerol).

#### Spinal cord

After perfusions, 1 cm of the cervical spinal cord (SC) was carefully removed from each mouse and post-fixed for 2 h at 4°C in 1% PFA solution. After washes, tissues were placed in 30% sucrose in PB at 4°C until completely submerged (~2 days) and then cryopreserved at −80°C. On the day of sectioning, SCs were embedded in optimal cutting temperature compound (Tissue-Tek) and cut longitudinally at −20°C into 14 μm sections using a Leica CM 1860 cryostat. Sections were immediately placed on SuperFrost Plus glass slides (Fisher; Pittsburgh, PA) and immunostained as described above for SN.

### Image analysis

Confocal images of teased SNs and the dorsal funiculus of the SC were acquired with a Zeiss LSM 710 Microscope using a 40x oil objective. Identical settings were maintained to capture images from control and mutant samples. The representative immunofluorescence images shown are maximal intensity projections from Z-stacks with an interval of 0.4 μm. For quantification of nodal intensities, three z-stack images were taken for each mouse. Then using paranodal markers to identify nodal boundaries, nodal areas were selected in order to measure area, intensity, and integrated density using ImageJ software (NIH; Bethesda, MD). A minimum of 50 nodes for each nodal marker per tissue were quantified. In addition, 10 background readings were taken of non-nodal areas within each image. This information was used to calculate the corrected fluorescence for each node = integrated density − (area of selected node × mean fluorescence of background readings) (McCloy et al., 2014).

### Immunoblotting

SNs and SCs were collected from anesthetized *CreER;NF*^*fx*/−^ and controls at various timepoints post-tamoxifen and stored at −80°C until processed. Extractions and immunoblotting were carried out as previously described (Thaxton et al., 2011). The only modifications include using IR-conjugated secondary antibodies (1:10,000 for 1 h) and imaging the membranes with an Odyssey scanner (LI-COR). Image Studio was used for intensity quantitation of bands relative to the loading control, α-tubulin.

### Quantitative PCR

SCs were collected from anesthetized *CreER;NF*^*fx*/−^ and controls at various timepoints post-tamoxifen and flash-frozen in liquid nitrogen. Tissues were stored at −80°C until total RNA was isolated using the TRIzol Plus RNA Purification System (Ambion). RNA concentrations were determined by absorbance at 260 nm. 2 μg of RNA was treated with RNase-free DNase and converted to cDNA using SuperScript II reverse transcriptase (Invitrogen). Quantitative real-time PCR (qPCR) was performed with an Applied Biosystems 7900HT. Each qPCR was analyzed in duplicate and contained in a final volume of 10 μl: 25 ng of cDNA, each primer at 150 nM, and 5 μl of 2x SYBR Green PCR Master Mix (Applied Biosystems). Results were evaluated by the comparative cycle number at threshold (CT) method using cyclophilin as the invariant housekeeping gene. Primers used for qPCR were designed based on Basak et al. (2007) or using NCBI Primer-BLAST, as follows: *Nfasc*^*NF186*^ FP-TCCAAGCATTCAGAATGAGCTG, RP-CCTGCTGTTGCGAGTCCAG; *Nfasc*^*NF155*^ FP-TCAGTGGAACCGAGTCTACTC, RP-ACCACCACCATCTCCAGCTTG; *Nfasc*^*Total*^ FP-GAAGCTAAAGGCAACCCCGC, RP-TTGTTCCGGGCAAAGCACTG.

### *In vivo* recordings

At various timepoints post-tamoxifen, *CreER;NF*^*fx*/−^ and controls were anesthetized by continuous isoflurane (5% aerosolized) for *in vivo* electrophysiological recordings using a Nicolet Teca Synergy portable neurological system (Natus Neurology Inc., Middleton, WI). During recordings, each mouse's temperature was maintained between 33 and 34°C using a warming lamp. Once fully anesthetized, nerve conduction velocities (NCV) and amplitudes were recorded from the tail and sciatic nerve as previously reported (Oh et al., 2010; Shi et al., 2014). Briefly, tail recordings were achieved by applying an electrical stimulus (0.02 ms, 4 mA) at the base of the tail and recording 30 mm distally. To record from the SN, the recording electrodes were placed in the dorsum of the foot, and two separate recordings were made. First, a stimulus was given at the ankle (0.02 ms, 3 mA) and then at the sciatic notch (0.02 ms, 4 mA). For each trace, amplitude was measured as the height of the peak, and NCV was calculated by the distance divided by the latency. In order to determine the sciatic NCV, the distance between the notch and ankle divided by the difference between the notch and ankle latencies was used. After the recordings, each mouse was monitored as it recovered from the anesthesia which took <1 min. As some minor nerve damage has been reported to occur through electrode placement (Schulz et al., 2014), NCV studies were limited to the left SN, so that the SN on the right side could be used for further analyses.

### Electron microscopy

*CreER;NF*^*fx*/−^ and controls at the specified timepoints post-tamoxifen were anesthetized and perfused intracardially using a peristaltic pump with saline for 10 min followed by a 2.5% glutaraldehyde:4% PFA PB solution for 30 min. After perfusion, the whole animal was submerged in fixative for at least 2 weeks before the SC and SN were dissected. The tissues were cut into 1 mm square pieces and left in sodium cacodylate buffer overnight. Then the samples were: (1) rinsed with buffer; (2) incubated in 2% OsO4 for 1 h; (3) rinsed with buffer; (4) dehydrated in ethanol (30–100%); (5) incubated in 100% propylene oxide (PO) for 1 h, 2PO:1Poly/Bed resin overnight, then 1PO:2Poly/Bed for 6 h, and finally in 100% Poly/Bed resin for 36 h; and (6) embedded in resin blocks at 55°C for 36 h. Once in blocks, the samples were submitted to the UTHSCSA Electron Microscopy Lab and processed as previously described (Green et al., 2013). Samples were imaged on a JEOL 1230 transmission electron microscope using an Advanced Microscopy Techniques camera and software (Woburn, MA). For quantification of healthy axons, 20 images were taken at 5000x for each tissue. From the images, a minimum of 200 axons for SN and 500 axons for SC were scored for each mouse.

### Data analysis

All data are presented as the mean ± SEM. Relative values were arithmetically adjusted to yield a unit of 1 for the control group at each timepoint. When only a single timepoint was used, statistically significant differences between *CreER;NF*^*fx*/−^ mice and controls were determined using student's *T*-tests. When comparing multiple timepoints across genotypes, two-way ANOVA were performed followed by Tukey's multiple comparison analyses. Statistic differences are represented by ^*^(*P* < 0.05), ^**^ (*P* < 0.01),^***^ (*P* < 0.001) with black asterisks indicating differences between age-matched control and mutants; while colored asterisks signify differences among the mutant group at different ages (red for *SLICK-H-CreER;NF*^*fx*/−^ and blue for *Actin-CreER;NF*^*fx*/−^). All statistical tests were performed using GraphPad Prism6 software (San Diego, CA).

## Results

### Neurofascin 186 disappears gradually from the nodes after adult ablation

**Figure 1:**
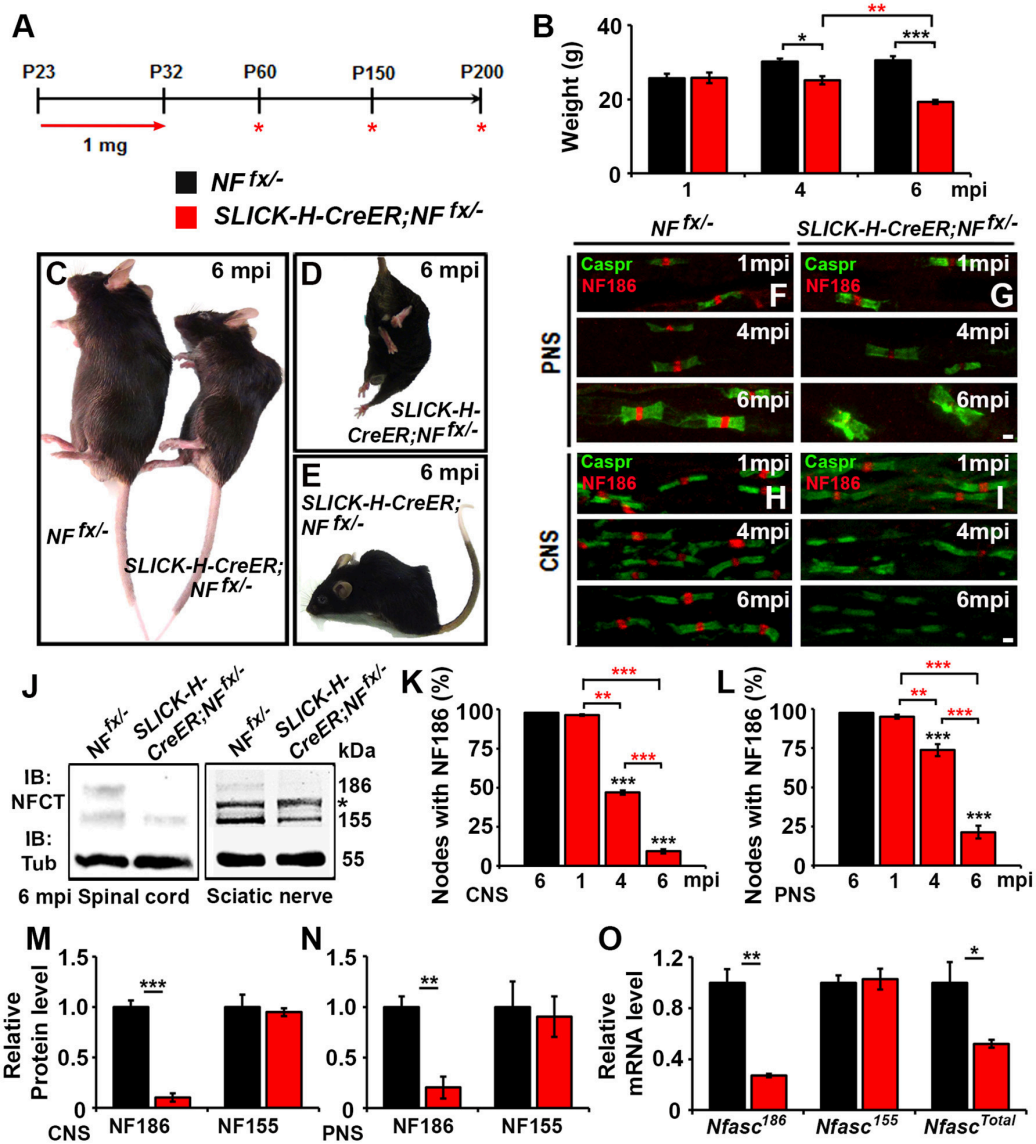
**Adult neuronal-specific ablation of neurofascin reveals NF186 durability at the node. (A)** Schematic representation of tamoxifen injections for *Nfasc*^*NF186*^ ablation from adult myelinated axons. *SLICK-H-CreER;NF*^*fx*/−^and *NF*^*fx*/−^ control mice were analyzed 1, 4, and 6 mpi. **(B)** Graph representing weight of *NF*^*fx*/−^ (black bar) and *SLICK-H-CreER;NF*^*fx*/−^(red bar) mice (males only, *n* = 5–8 mice /group). **(C–E)** Photographic depiction of 6 mpi *SLICK-H-CreER;NF*^*fx*/−^ mutant and *NF*^*fx*/−^ control mice after NF186 ablation by tamoxifen. **(F–I)** Immunostaining of 1, 4, 6 mpi teased SN fibers **(F,G)** and SCs **(H,I)** with antibodies against Caspr (green) and NF186 (red) from *NF*^*fx*/−^ and *SLICK-H-CreER;NF*^*fx*/−^mice. **(J)** Immunoblot analysis of SC and SN lysates from 6 mpi *NF*^*fx*/−^ and *SLICK-H-CreER;NF*^*fx*/−^mice with antibodies against NFCT and Tub. Asterisk shows size of a non-specific band present in the SN. **(K,L)** Quantification of the percentage of nodes with remaining NF186 at 1, 4, and 6 mpi in *NF*^*fx*/−^ and *SLICK-H-CreER;NF*^*fx*/−^ in SC or SN, respectively (*n* = 4–6/group). **(M,N)** Quantification of immunoblots relative to α-Tubulin from the SC or SN lysates, respectively (*n* = 3–4/group). **(O)** mRNA analysis of SC from 1 mpi *NF*^*fx*/−^ and *SLICK-H-CreER;NF*^*fx*/−^mice by qPCR with primers specific to NF186, NF155, or common to both isoforms of *Nfasc* (*n* = 3–4/group). All data are represented as mean ± SEM. Black asterisks indicate statistical differences between control and mutant; while red asterisks signify differences between timepoints among mutants. See also Movie 1. Scale bar, 2 μm.

To study the individual contribution of *Nfasc*^*NF*186^ to the long-term maintenance of the node, we crossed *Nfasc*^*fx*/−^ (*NF*^*fx*/−^) mice (Pillai et al., 2009) with Single Neuron Labeling with Inducible Cre-mediated Knockout *(SLICK-H-CreER)* mice, which utilize a *Thy1.2* promotor modified to be specifically expressed in neurons (Caroni, 1997; Young et al., 2008). *SLICK-H-CreER;NF*^*fx*/−^ and *NF*^*fx*/−^ control littermates were given tamoxifen injections for 10 consecutive days starting at P23 to specifically ablate NF186 from neurons after myelination was complete. These mice were examined at 1, 4, and 6 months of injection (mpi), as *SLICK-H-CreER;NF*^*fx*/−^ did not survive until 7 mpi (Figure 1A). Although adult loss of NF186 ultimately led to an ataxic phenotype and shortened lifespan, *SLICK-H-CreER;NF*^*fx*/−^ mice did not show a difference in body weight, gait, or tremor until 4 mpi which then enhanced rapidly (Figure 1B). By 6 mpi, *SLICK-H-CreER;NF*^*fx*/−^ mice displayed foot clasping, abnormal gait, partial hindlimb paralysis, and severe spine deformation (Figures 1C–E, Movie 1), which was never observed in tamoxifen injected littermate *NF*^*fx*/−^ controls. In accordance with the timing of the phenotype, immunostaining of sciatic nerve (SN) fibers and spinal cord (SC) slices before 4 mpi showed no significant loss of NF186 in *SLICK-H-CreER;NF*^*fx*/−^ mice (Figures 1F–I). In SC, quantification analysis revealed 53.1 ± 1.4% of nodes had no detectable levels of NF186 at 4 mpi, which grew to 90.6 ± 1.3% by 6 mpi (Figure 1K); while 26.3 ± 3.9% of SN nodes had lost NF186 by 4 mpi, which was increased to 78.6 ± 4.1% by 6 mpi (Figure 1L). Further immunoblotting of SC and SN (Figure 1J, asterisk indicates non-specific band in SN) with an antibody to the C-terminus of Nfasc (NFCT, detects both NF155 and NF186) shows a significant reduction in NF186 by 6 mpi in *SLICK-H-CreER;NF*^*fx*/−^ mice compared to *NF*^*fx*/−^ mice (Figures 1M,N). However, there was no change in the myelinating glia NF155 isoform in either CNS or PNS (Figures 1J,M,N). While protein levels of NF186 appear to be stable before 4 mpi and then reduced to 10% by 6 mpi in SC (Figures 1K,M), qPCR analyses show *Nfasc*^*NF186*^ mRNA levels to be significantly down by 73 ± 1.3% as early as 1 mpi (Figure 1O). In addition, there is no change in *Nfasc*^*NF155*^ mRNA levels alone using a primer specific to the FN3 domain of NF155; while a 48 ± 3.2% reduction is detected in *Nfasc*^*Total*^ mRNA using a primer specific to the FN1 domain present in both NF155 and NF186 (Figure 1O). Together, these results reveal that complete turnover of NF186 at mature nodes takes up to 6 mpi after its ablation and that NF186 is highly stable once incorporated into the nodal complex.

### Adult ablation of neurofascin 186 leads to progressive nodal destabilization in both the PNS and CNS

**Figure 2:**
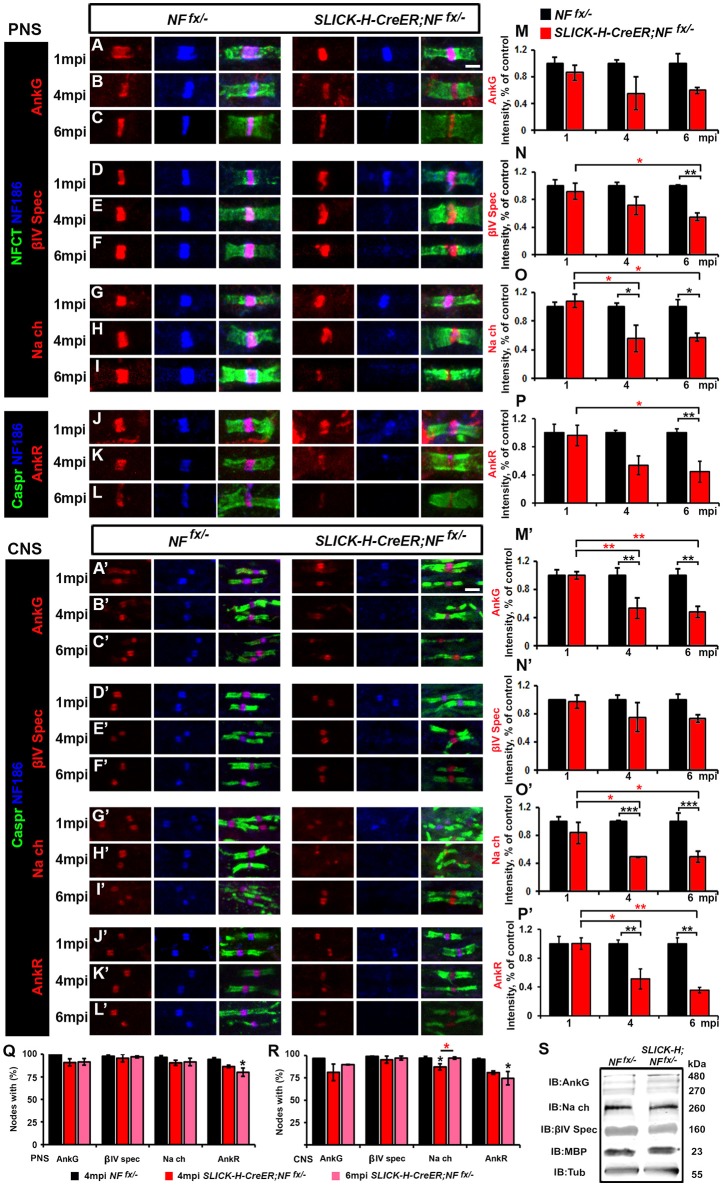
**Adult ablation of NF186 alone causes progressive nodal destabilization. (A–I)** SN fibers from 1, 4, and 6 mpi age-matched *NF*^*fx*/−^ and *SLICK-H-CreER;NF*^*fx*/−^littermate mice were immunostained with antibodies against NF186 (blue) and NFCT (green) in combination with either of the following proteins: AnkG **(A–C)**, βIV Spec **(D–F)** or pan-Na_V_**(G–I)** (red). **(J–L)** PNS fibers from 1, 4, and 6 mpi age-matched *NF*^*fx*/−^ and *SLICK-H-CreER;NF*^*fx*/−^littermate mice were immunostained with antibodies against NF186 (blue), Caspr (green), and AnkR (red). **(M–P)** Graphs representing intensity quantification of AnkG, βIV Spec, pan-Na_V_, and AnkR respectively in the PNS nodal area of 1, 4, and 6 mpi *SLICK-H-CreER;NF*^*fx*/−^ (red bars) normalized to aged-matched *NF*^*fx*/−^ control values (black bars). **(A'–L')** SCs from 1, 4, and 6 mpi age-matched *NF*^*fx*/−^ and *SLICK-H-CreER;NF*^*fx*/−^littermate mice were immunostained with antibodies against NF186 (blue) and Caspr (green) in combination with either of the following proteins: AnkG **(A'–C')**, βIV Spec **(D'–F')**, pan-Na_V_ **(G'–I')**, and AnkR **(J'–L')** (red). **(M'–P')** Graphs representing intensity quantification of AnkG, βIV Spec, pan-Na_V_, and AnkR respectively in the SC nodal area of 1, 4, and 6 mpi *SLICK-H-CreER;NF*^*fx*/−^ (red bars) normalized to aged-matched *NF*^*fx*/−^ control values (black bars). **(Q–R)** Quantification of the percentage of PNS **(Q)** and CNS **(R)** nodes with remaining AnkG, βIV-Spectrin, pan Na_V_, and AnkR at 4 and 6 mpi in *SLICK-H-CreER;NF*^*fx*/−^ mice compared to *NF*^*fx*/−^ controls. **(S)** Immunoblot analysis of SC lysates from 6 mpi *NF*^*fx*/−^ and *SLICK-H-CreER;NF*^*fx*/−^ mice with antibodies against AnkG, panNa_v_, βIV-Spec, MBP and tubulin (Tub; as a loading control). All data are represented as mean ± SEM (*n* = 4–6 mice/group). Black asterisks indicate statistical differences between control and mutant; while red asterisks signify differences between timepoints among mutants. Scale bar, 2 μm.

To determine the consequences of the slow loss of NF186 on the maintenance and stability of the nodal region, teased SNs from 1, 4, and 6 mpi *NF*^*fx*/−^ and *SLICK-H-CreER;NF*^*fx*/−^ were triple immunostained with antibodies against NF186, NFCT which simultaneously detects paranodal NF155 and nodal NF186, and either AnkG, βIV Spec, or pan-Na_V_ (Figures 2A–I). Additionally, PNS nerves were immunostained with antibodies against NF186, Caspr, and AnkR (Figures 2J–L), an additional nodal Ankyrin protein which was recently reported to be highly localized at the node in AnkG-deficient nodes (Ho et al., 2014). At 1 mpi, when NF186 was still present at the nodes without any appreciable loss, there were no changes in either the localization or intensity of AnkG (Figure 2A), βIV Spectrin (βIV Spec) (Figure 2D), pan-Na_V_ channels (Figure 2G), or AnkR (Figure 2J). By 4 mpi when NF186 was undetectable in 26.3% of nodes and barely detectable in the remaining nodes of *SLICK-H-CreER;NF*^*fx*/−^ (Figures 2B,E,H,K), scoring nodes which had loss of AnkG, βIV Spec, pan-Na_V_, and AnkR revealed only an 8.4, 3.7, 8.5, and 10.5% reduction from control values, respectively (Figure 2Q). Although there was essentially no loss of other nodal proteins by 4 mpi, reductions in the intensity of AnkG, βIV Spec, pan-Na_V_, and AnkR were noticeable but only significant for pan-Na_V_ in *SLICK-H-CreER;NF*^*fx*/−^ compared to *NF*^*fx*/−^ (Figures 2B,E,H,K,M–P). By 6 mpi when NF186 was below levels of detection in the majority of *SLICK-H-CreER;NF*^*fx*/−^ nodes, there is still no substantial loss of AnkG (8.4%), βIV Spec (0.6%), pan-Na_V_ channels (5.2%) or AnkR (14.3%) detected from *NF*^*fx*/−^ controls, although the loss of AnkR reached significance (Figure 2R). However at 6 mpi, the intensity of βIV Spec, pan-Na_V_, and AnkR was significantly lower in *SLICK-H-CreER;NF*^*fx*/−^ compared to *NF*^*fx*/−^ (Figures 2F,I) with intensities of 45.4, 42.6, and 55.5%, respectively (Figures 2N,O). While even at 6 mpi, AnkG was not significantly lower in *SLICK-H-CreER;NF*^*fx*/−^ compared to *NF*^*fx*/−^ (Figures 2C,M), there was a strong trend (40.3%, *p*-value: 0.089) toward a decrease. Interestingly, when small amounts of NF186 were detected at the destabilizing nodes, AnkG, AnkR, pan-Na_V_, or βIV Spec always appeared to co-localize with NF186 suggesting that NF186 is the primary stabilizer of the nodal components. Together, these data indicate that NF186 is highly stable at the node and that the presence of NF186 is critical for the long-term stability of the nodal complex.

To further study NF186 role in nodal maintenance in the CNS, SCs from 1, 4, and 6 mpi *NF*^*fx*/−^ and *SLICK-H-CreER;NF*^*fx*/−^were also immunostained with antibodies against NF186, Caspr, and either AnkG (Figures 2A'–C'), βIV Spec (Figures 2D'–F'), pan-Na_V_ channels (Figures 2G'–I'), and AnkR (Figures 2J'–L'). As was seen in the PNS, at 1 mpi before NF186 is turned over, there were no changes in CNS in either localization or intensity of AnkG (Figure 2A'), βIV Spec (Figure 2D'), pan-Na_V_ (Figure 2G'), or AnkR (Figure 2J'). At 4 mpi when NF186 is undetectable in over half the nodes of *SLICK-H-CreER;NF*^*fx*/−^ SCs, scoring nodes which had loss of AnkG, βIV Spec, pan-Na_V_, or AnkR revealed only slight reductions at 15.6, 3.2, 9.2, and 8.3% respectively (Figure 2R). While very little loss of other nodal proteins was observed from nodes lacking NF186, there were reductions in the intensity of remaining proteins in *SLICK-H-CreER;NF*^*fx*/−^ compared to *NF*^*fx*/−^ SCs at 4 mpi (Figures 2B',E',H',K'). Quantification analysis at 4 mpi shows a significant reduction of nodal AnkG, pan-Na_V_, and AnkR intensity by 46.4, 51.1, and 48.9% compared to controls levels (Figures 2M',O',P'), but not in βIV Spec which was reduced by only 24.9% (Figure 2N'). At 6 mpi when NF186 is undetectable in 90.6% of *SLICK-H-CreER;NF*^*fx*/−^ nodes in SC, there is a slight but significant loss of AnkG (7.1%); however, no significant loss of either βIV Spec, pan-Na_V_, or AnkR was detected from control values (Figure 2R). Consistent with the changes in PNS at 6 mpi, the intensity of AnkG, pan-Na_V_, and AnkR are significantly lower in *SLICK-H-CreER;NF*^*fx*/−^ CNS compared to *NF*^*fx*/−^ (Figures 2C',I',L') with reductions of 51.4, 50.8, and 64.5% respectfully (Figures 2M'–P'). While even at 6 mpi, βIV was not significantly lower in *SLICK-H-CreER;NF*^*fx*/−^ SC (Figures 2D',N'), there was a strong trend (26.4%, *p*-value: 0.065) toward a decrease compared to *NF*^*fx*/−^. Overall, these data indicate that once the nodal complex is assembled, it remains highly stable for long periods of time and that loss of NF186 in adults causes a gradual destabilization of pan-Na_V_, AnkR/G and βIV Spec from the nodes in the CNS. Despite the pronounced decrease of NF186 in *SLICK-H-CreER;NF*^*fx*/−^ SC, there were no changes in the total protein levels of AnkG, βIV Spec, pan-Na_V_ channels, or Myelin Basic Protein (MBP) normalized to tubulin compared to *NF*^*fx*/−^ controls (Figure 2S). Together these data suggest that the steady state levels of other nodal proteins are not significantly affected, but instead the localization of these proteins is compromised due to destabilization of the NF186-deficient nodal complex and dispersal of these proteins along axons.

### Paranodal loss alone does not affect nodal stability

**Figure 3:**
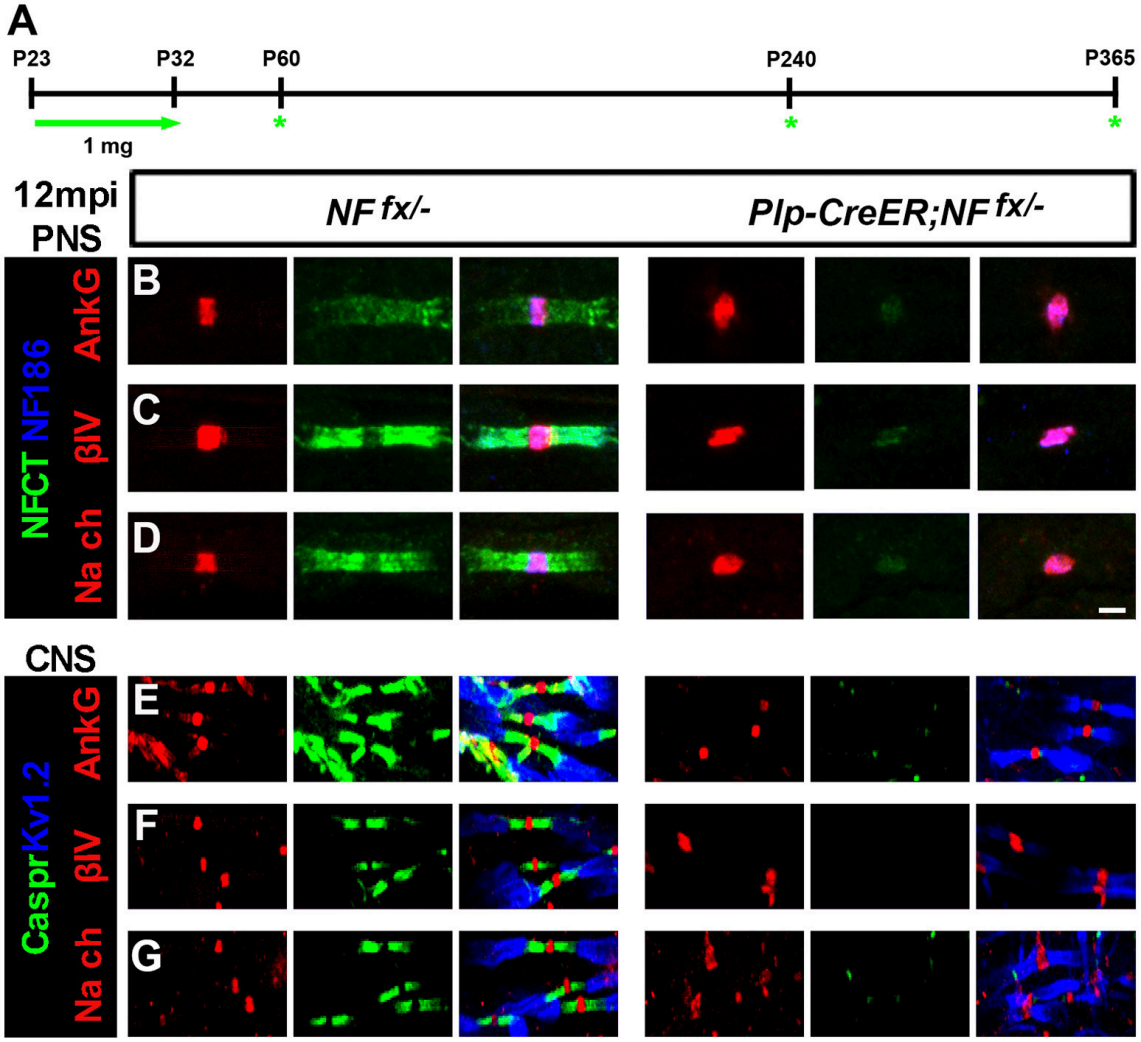
**Nodal proteins remain stable despite disruption in the flanking paranodal Axo-glial junctions. (A)** Schematic representation of tamoxifen injections for *Nfasc*^*NF155*^ ablation from adult myelinating glial cells. *Plp-CreER;NF*^*fx*/−^and *NF*^*fx*/−^ control mice were analyzed 1, 7, and 12 mpi. **(B–D)** SN fibers from 12 mpi *NF*^*fx*/−^ and *Plp-CreER;NF*^*fx*/−^ littermate mice were immunostained with antibodies against NF186 (blue) and NFCT (green) in combination with either of the following proteins: AnkG **(A)**, βIV Spec **(B)** or pan Na_V_ **(C)** (red). **(E–G)** SCs from 12 mpi *NF*^*fx*/−^ and *Plp-CreER;NF*^*fx*/−^ littermate mice were immunostained with antibodies against K_V_1.2 (blue) and Caspr (green) in combination with either of the following proteins: AnkG **(D)**, βIV Spec **(E)** or pan Na_V_ **(F)** (red). Scale bar, 2 μm. The green asterisks are part of the schematic indicating when mice were studied as was used in Figures 1A, 4A.

In order to thoroughly elucidate the role of paranodal region in the life-long stability of nodes, we chose to genetically ablate paranodal NF155 individually using a tamoxifen inducible myelinating glia Cre (*Plp-CreER*) (Doerflinger et al., 2003) and in combination with NF186. Although, we previously showed that nodes persist for up to 3 mpi in *Plp-CreER*;*NF*^*fx*/*fx*^ when NF155 is ablated during adolescence after myelination is complete (Pillai et al., 2009), in this report we extended these studies until 12 mpi (Figure 3). *Plp-CreER*;*NF*^*fx*/−^ mice along with *NF*^*fx*/−^ control littermates were given tamoxifen injections for 10 days from P23-32 as was done previously. These mice were examined at 1, 7, and 12 mpi (Figure 3A). Even at 12 mpi, the *Plp-CreER*;*NF*^*fx*/−^ mice maintained average body weights (23.3 ± 1.14 g for females compared to *NF*^*fx*/−^ females at 22.0 ± 1.29 g, *n* = 3 mice/group); however, the mutant mice showed slight tremors and abnormal gait. In addition, *in vivo* electrophysiological recordings from the SN showed a significant reduction in NCV at 12 mpi in *Plp-CreER*;*NF*^*fx*/−^ mice (19.8 ± 1.85 m/s) compared to *NF*^*fx*/−^ littermate controls (34.4 ± 1.26 m/s, *n* = 4 mice/group).

To determine the consequences of paranodal NF155 ablation on nodal maintenance, teased SNs and sectioned SCs from 1, 7, and 12 mpi *NF*^*fx*/−^ and *SLICK-H-CreER;NF*^*fx*/−^ were triple immunostained with antibodies against NF186 and NFCT or K_V_1.2 and Caspr with either AnkG, βIV Spec, or pan-Na_V_. Even 1 year after tamoxifen, AnkG, βIV Spec, and pan-Na_V_ channels were still clustered at the nodes in the absence of paranodes in PNS (Figures 3B–D, only 12 mpi shown) and CNS (Figures 3E–G); however, the nodal region becomes more diffuse in *Plp-CreER*;*NF*^*fx*/−^ than in *NF*^*fx*/−^. These results show that the paranodal region is not individually required for nodal maintenance but may play a supportive role with other mechanisms at the node.

### Simultaneous loss of nodal NF186 and paranodal NF155 dramatically accelerates nodal destabilization

**Figure 4:**
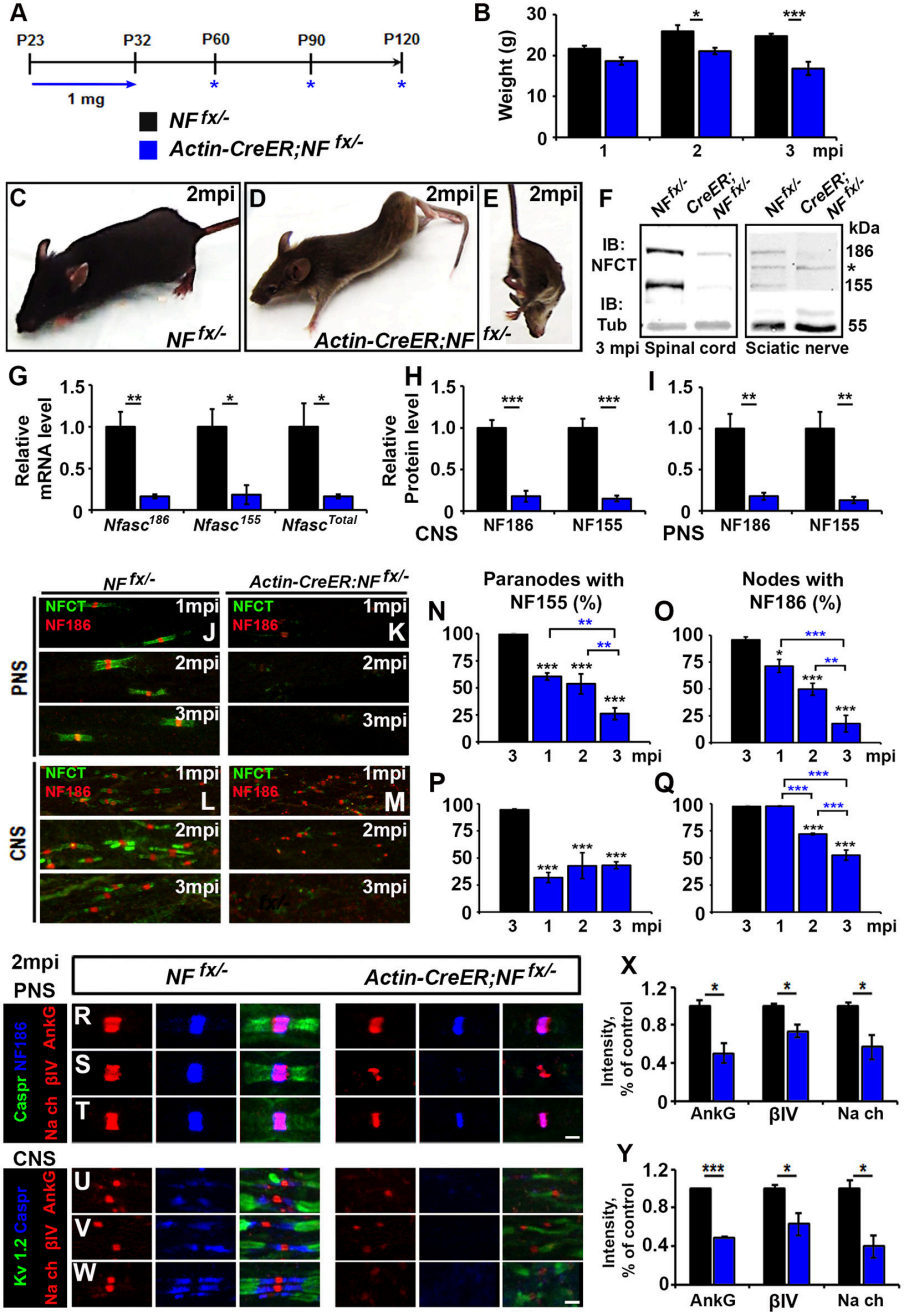
**Adult ablation of paranodal NF155 in combination with nodal NF186 accelerates nodal destabilization. (A)** Schematic representation of tamoxifen injections for simultaneous NF155 and NF186 ablation from adults. Tamoxifen was injected starting at P23 for 10 consecutive days to *Actin-CreER;NF*^*fx*/−^and *NF*^*fx*/−^control mice, which were analyzed 1, 2, and 3 mpi. **(B)** Graph representing body weight of *NF*^*fx*/−^ (black bar) and *Actin-CreER;NF*^*fx*/−^ (blue bar) mice (males only, *n* = 4–6 mice/group). **(C–E)** Photographic depiction of 2 mpi **(C–E)** Photographic depiction of 2 mpi *NF*^*fx*/−^ control **(C)** and Actin-CreER;*NF*^*fx*/−^ mutant **(D–E)** mice after NF155/186 ablation by tamoxifen. **(F)** Immunoblot analysis of SC and SN from 3 mpi *NF*^*fx*/−^ and *Actin-CreER;NF*^*fx*/−^ mice with antibodies against NFCT and Tub. Asterisk shows size of a non-specific band present in SN. **(G)** mRNA analysis of SC from 3 mpi *NF*^*fx*/−^ and *Actin-CreER;NF*^*fx*/−^mice by qPCR with primers specific to NF186, NF155, or common to both isoforms of *Nfasc* (*n* = 4/group). **(H,I)** Quantification of immunoblots relative to alpha-Tubulin in SC or SN, respectively (*n* = 3–4/group). **(J–M)** Immunostaining of 1, 2, and 3 mpi teased SN fibers **(G,H)** and SCs **(I,J)** with antibodies against NFCT (green) and NF186 (red) from *NF*^*fx*/−^ and *Actin-CreER;NF*^*fx*/−^ mice. **(N–Q)** Quantification of the percentage of paranodes with remaining NF155 **(N,P)** and of nodes with remanding NF186 **(O,Q)** at 1, 2, and 3 mpi in *NF*^*fx*/−^ and *Actin-CreER;NF*^*fx*/−^ in SN or SC, respectively (*n* = 4–6/group). **(R–T)** SN fibers from 2 mpi *NF*^*fx*/−^ and *Actin-CreER;NF*^*fx*/−^ littermate mice were immunostained with antibodies against NF186 (blue) and Caspr (green) in combination with either of the following proteins: AnkG, βIV Spec or pan-Na_V_(red). **(U–W)** SC sections from 2 mpi *NF*^*fx*/−^ and *Actin-CreER;NF*^*fx*/−^ littermate mice were immunostained with antibodies against K_V_1.2 (green) and Caspr (blue) in combination with either of the following proteins: AnkG, βIV Spec or pan-Na_V_ (red). **(X,Y)** Graphs representing intensity quantification of AnkG, βIV Spec or pan-Na_V_in the SN and SC respectively (*n* = 3–4/group). All data are represented as mean ± SEM. See also Movie 2. Scale bar, 2 μm. Black asterisks indicate statistical differences between control and mutant; while blue asterisks signify differences between timepoints among mutants.

To address the combined contribution of neuronal NF186 and glial NF155 at the nodal regions, we simultaneously ablated both isoforms using a tamoxifen inducible ubiquitous Cre (*ActinCre-ER*) (Hayashi and McMahon, 2002). NF^*fx*/−^ mice were crossed to *Actin-CreER* mice and given tamoxifen injections from P23-32 as was done to ablate NF186 and NF155 individually. Since *Actin-CreER*;*NF*^*fx*/−^ failed to survive past 3 mpi, they were examined at 1, 2, and 3 mpi (Figure 4A). The difference between *Actin-CreER*;*NF*^*fx*/−^ mice and their control littermates was evident by the significant reduction in body weight starting at 2 mpi (Figures 4B–D). Mice with ablation of both NF155/NF186 showed an extreme rapidly progressing phenotype (Figures 4D–E, pictured at 2 mpi), which started with foot clenching and abnormal gait at 1 mpi and resulted in complete hindlimb paralysis by 3 mpi (Movie 2). To confirm NF155 and NF186 ablation by tamoxifen, immunoblots were performed at 3 mpi in SC and SN (Figure 4F), which reveal a significant reduction of more than 80% in the protein levels of both NF155 and NF186 in *Actin-CreER*;*NF*^*fx*/−^ SCs and SNs compared to controls (Figures 4H,I). In addition to the reduction in protein level, an over 80% loss of *Nfasc* mRNA was confirmed in SC at 3 mpi by qPCR analyses with primers specific to NF186, NF155 or common to both (Figure 4G). Further SNs and SCs were examined from 1 to 3 mpi with immunostaining using antibodies against NFCT and NF186. While paranodal NF155 and nodal NF186 were present at all timepoints in NF^*fx*/−^ mice, by 1 mpi NF155 was undetectable in 60.5 ± 3% of paranodes in the PNS and 68.2 ± 4.7% in the CNS of *Actin-CreER*;*NF*^*fx*/−^ mice (Figures 4J–M). Despite the loss of NF155, NF186 remains at most nodes 1 mpi in *Actin-CreER*;*NF*^*fx*/−^, albeit at lower intensity (Figures 4K,M). By 2 mpi, nodes with remaining NF186 were seldom seen without some traces of NF155 in at least 1 flanking paranodal domain (Figures 4K,M), indicating nodes where NF155 was completely ablated from each flanking paranode had already lost NF186. By 3 mpi, nodes or paranodes were rarely observed in the CNS or PNS (Figures 4K–M). However, the percentage of remaining paranodes with NF155 and nodes with NF186 does not fully reflect this loss as it is over the detectable nodes and does not include axons where nodes and paranodes could no longer be detected (Figures 4N–Q). Taken together, these results highlight the differential turnover rate of paranodal NF155 and nodal NF186. When both Nfasc are simultaneously lost, glial NF155 is depleted first which then permits an accelerated turnover of neuronal NF186 at the mature nodes. Compared to when NF186 is individually ablated, combined ablation lead to a quicker loss of NF186 at the nodes of Ranvier revealing a supportive role of the paranodal complex in nodal maintenance in both the PNS and CNS.

To determine the consequences of combined loss of NF186 and NF155 on the maintenance and stability of the node, SNs from 1, 2 and 3 mpi *NF*^*fx*/−^ and *Actin-CreER*;*NF*^*fx*/−^ were triple immunostained with antibodies against NF186, paranodal Caspr, and either AnkG (Figure 4R), βIV Spec (Figure 4S), or pan-Na_V_ channels (Figure 4T). At 2 mpi despite paranodal NF155 loss, as long as some NF186 remained at the node, all other nodal proteins were intact; however, their intensity was significantly reduced compared to controls (Figure 4X). When nodal NF186 was no longer detectable, other nodal proteins began to diffuse but could be held temporarily by remaining axo-glial paranodal junctions as seen for βIV Spec in Figure 4S. To determine the combined role on nodal stability in the CNS, SCs from 1, 2, and 3 mpi *NF*^*fx*/−^ and *Actin-CreER*;*NF*^*fx*/−^ were triple immunostained with antibodies against juxtaparanodal K_V_1.2, paranodal Caspr, and either AnkG (Figure 4U), βIV Spec (Figure 4V), or pan-Na_V_ channels (Figure 4W). As in PNS, all nodal markers were present at 2 mpi, but the intensity of all these nodal proteins was significantly reduced (Figure 4Y). By 3 mpi in both PNS and CNS, AnkG, βIV Spec, and pan-Na_V_ channels were no longer clustered at the nodes that lacked NF186 and instead were either absent or diffusely distributed around the nodal area (data not shown). Together, the combined loss of NF186 and NF155 reveals that the paranodal complex functions to prevent the turnover of the nodal NF186 and thus helps to maintain the stability of the nodal complex.

### Ablation of NF186 alone and with NF155 leads to reduced nerve conduction

**Figure 5:**
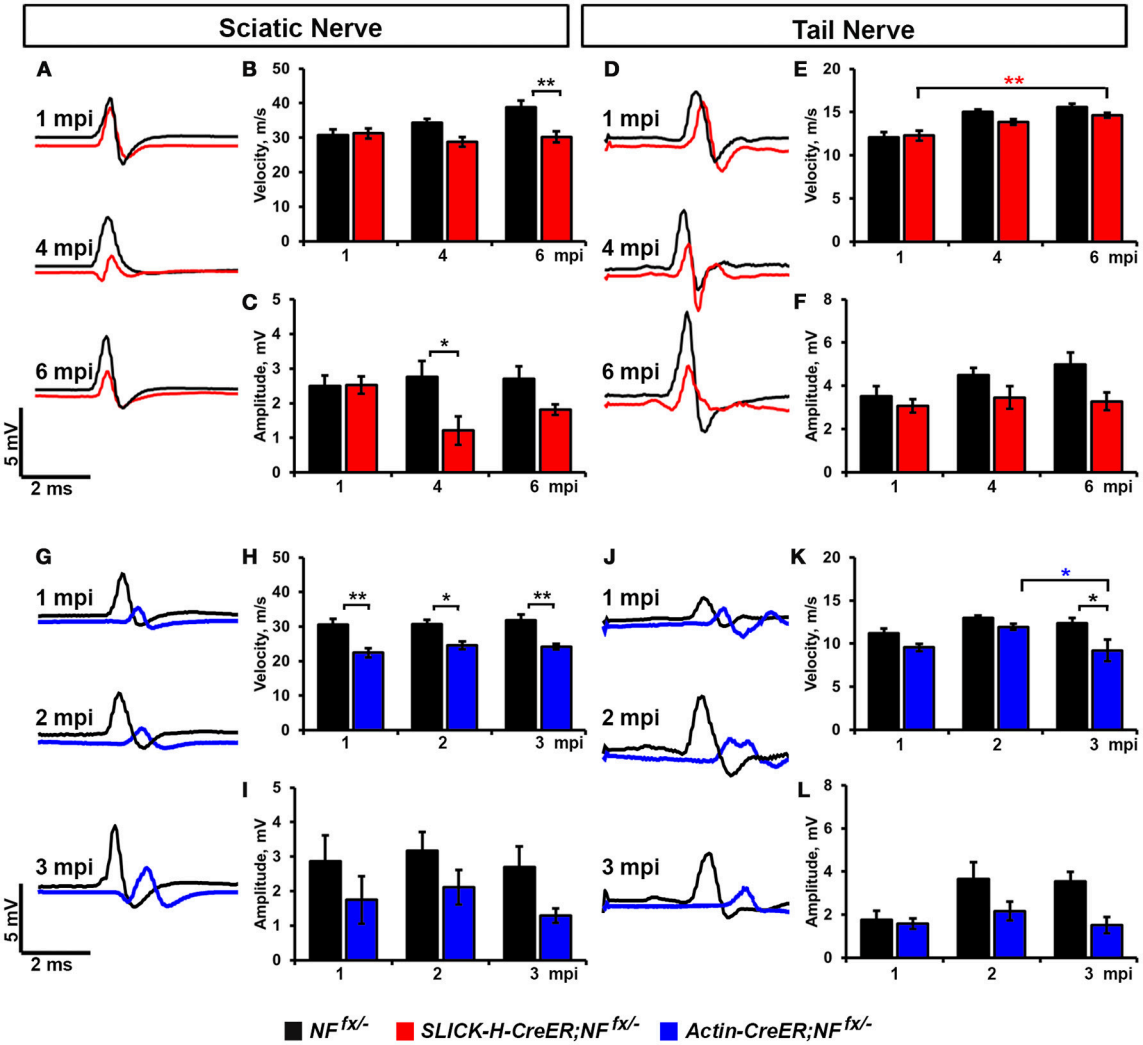
**Destabilization of nodes leads to reduced nerve conduction velocity. (A–C)** Representative electrophysiological profiles **(A)** of Compound Action Potentials (CAPs) from SNs of 1, 4, and 6 mpi *NF*^*fx*/−^ (black line) and *SLICK-H-CreER;NF*^*fx*/−^ (red line) with quantification of NCV **(B)** and amplitude **(C)**. **(D–F)** Representative electrophysiological profiles **(D)** of CAPs from tails of 1, 4, and 6 mpi *NF*^*fx*/−^ and *SLICK-H-CreER;NF*^*fx*/−^ with quantification of NCV **(E)** and amplitude **(F)**. **(G–I)** Representative electrophysiological profiles **(G)** of CAPs from SNs of 1, 2, and 3 mpi *NF*^*fx*/−^ (black line) and *Actin-CreER;NF*^*fx*/−^ (blue line) with quantification of NCV **(H)** and amplitude **(I)**. **(J–L)** Representative electrophysiological profiles **(J)** of CAPs from tails of 1, 2, and 3 mpi *NF*^*fx*/−^ and *Actin-CreER;NF*^*fx*/−^ with quantification of NCV **(K)** and amplitude **(L)**. All data are represented as mean ± SEM (*n* = 7–10 mice/group). Black asterisks indicate statistical differences between control and mutant; while colored asterisks signify differences between timepoints among the mutant group (red for *SLICK-H-CreER;NF*^*fx*/−^ and blue for *Actin-CreER;NF*^*fx*/−^).

As significant reductions in intensity of pan-Na_V_ were seen after ablation of nodal NF186 alone or in combination with paranodal NF155 in both PNS and CNS, we next wanted to determine how loss of nodes and paranodes impacted the electrophysiological properties of myelinated axons over time. Thus, we performed *in vivo* electrophysiological recordings from the SN and the tail of *SLICK-H-CreER;NF*^*fx*/−^ and *Actin-CreER;NF*^*fx*/−^ mice (Figure 5). While no changes in either conduction velocity or CAP amplitude were seen in *SLICK-H-CreER;NF*^*fx*/−^ mice compared to *NF*^*fx*/−^ mice at 1 mpi, significant changes were observed in later timepoints, which correspond with loss of NF186 from the nodes (Figures 5A–F). In the SN, NCV was reduced at 4 mpi and significantly lower by 6 mpi in *SLICK-H-CreER;NF*^*fx*/−^ compared to *NF*^*fx*/−^ (Figures 5A,B). However these changes in NCV were not observed in the tail between the littermate controls and *SLICK-H-CreER;NF*^*fx*/−^ at any timepoint and the only significant change noted was a slight increase in NCV with age in both groups (Figures 5D,E). While there were trends toward a decrease in CAP amplitude in both SN and tail of *SLICK-H-CreER;NF*^*fx*/−^ compared to *NFf*^*x*/−^, neither were significant by 6 mpi (Figures 5C,F). Overall, these electrophysiological measurements indicate an individual role for NF186 in maintaining sufficient levels of Na_V_ channels at nodes in order to propagate action potentials and maintain proper conduction velocity along myelinated axons.

Although no differences in nerve conduction were found before 4 mpi when NF186 was ablated individually, significant decrease in conduction velocity were already apparent at 1 mpi in *Actin-CreER;NF*^*fx*/−^when NF186 and NF155 were simultaneously ablated (Figures 5G–L). In SN, NCV was significantly reduced in *Actin-CreER;NF*^*fx*/−^ compared to *NF*^*fx*/−^ at all timepoints (Figures 5G,H). While in tail, a significant reduction in NCV was detected at 3 mpi between *Actin-CreER;NF*^*fx*/−^and both the 3 mpi control group as well at the 2 mpi *Actin-CreER;NF*^*fx*/−^(Figures 5J,K). This depreciation in conduction velocity in PNS, corresponds with the turnover of NF186 at the nodes (Figure 4O). While no significant differences were seen in CAP amplitude in *Actin-CreER;NF*^*fx*/−^ compared to control, trends toward a decrease were apparent in both SN and tail at 3 mpi (Figures 5G,I,J,L). Taken together, these studies further emphasize a supporting role for flanking paranodal regions along with NF186 in preserving the integrity of the nodal complex and thereby sustaining the electrical properties of myelinated axons.

### Destabilization of nodes in adults leads to ultrastructural changes and degeneration of myelinated axons

**Figure 6:**
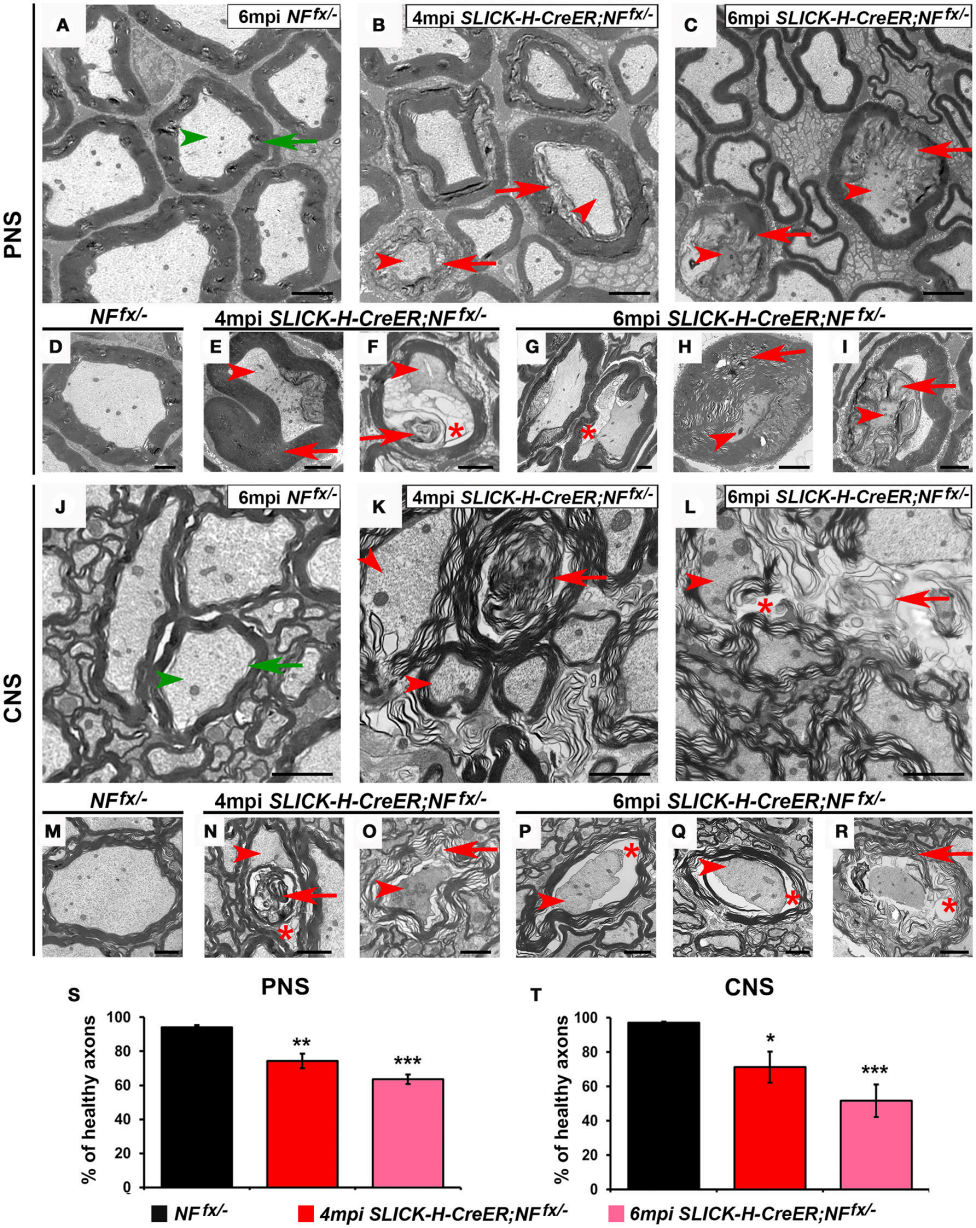
**Disorganization of nodes leads to degeneration of adult myelinated axons. (A–I)** Transmission electron microscopy (TEM) of cross sections from SNs of control *NF*^*fx*/−^ mice at 6 mpi **(A,D)** and *SLICK-H-CreER;NF*^*fx*/−^ mice at 4 mpi **(B,E,F)** and 6 mpi **(C,H,I)**. Green arrowheads point to normal axons, red arrowheads to axonal pathology, greens arrows to compact myelin, red arrows to abnormal myelin or myelin inclusions, and red asterisks to vacuoles within axons. **(J–R)** TEM of cross sections from SCs of control *NF*^*fx*/−^ mice at 6 mpi **(J,M)** and *SLICK-H-CreER;NF*^*fx*/−^ mice at 4 mpi **(K,N,O)** and 6 mpi **(L,P–R)**. **(S–T)** Quantification of healthy axons found in the cross sections of *NF*^*fx*/−^ mice (black bar) and *SLICK-H-CreER;NF*^*fx*/−^ (red and pink bars) SN and SC, respectively. A minimum of 200 axons were scored for SN and 500 axons for SC of each mouse. All data are represented as mean ± SEM (*n* = 4 mice/ group). Scale bar, 2 μm. Black asterisks indicate statistical differences between control and mutant.

Since adult ablation of NF186 either alone or in combination with NF155 lead to nodal disorganization and concomitant reduction in nerve conduction, we next examined myelinated axons for any ultrastructural defects that might have occurred over time. At 4 and 6 mpi, SNs and SCs from *NFfx*^/−^ and *SLICK-H-CreER;NF*^*fx*/−^ were processed for electron microscopy; and representative images are presented in Figure 6. The cross sections from control SNs and SCs show morphologically normal axons (green arrowhead) surrounded by tight myelin ensheathment (green arrows) as expected (Figures 6A,D,J,M). In contrast the SNs of 4 mpi *SLICK-H-CreER;NF*^*fx*/−^ reveal the presence of myelinated fibers with signs of axonal pathology or in severe cases axonal degeneration (Figures 6B,E,F). As axonal ruffling and shrinking occurred (red arrowheads), vacuoles (red asterisks) were created within the axons which appeared to be filled in by myelin (red arrows). By 6 mpi, most large and some smaller caliber axons were found to show signs of degeneration with abnormal myelin inclusion within the axons; however, normal small caliber axons were still prevalent (Figures 6C,G–I). In order to quantify these phenotypes, normal axons were scored from *NF*^*fx*/−^ and *SLICK-H-CreER;NF*^*fx*/−^ mice (Figure 6S). At both 4 mpi (25.6 ± 4.3%) and 6 mpi *SLICK-H-CreER;NF*^*fx*/−^ (36.4 ± 2.7%), mice with postnatal ablation of NF186 had more abnormal axonal morphologies in SNs compared to controls (5.9 ± 1.3%). Similar to what was observed in SNs, axonal pathology (arrowheads) and abnormal myelin structures (arrows) are seen in approximately 1 in 4 axons in *SLICK-H-CreER;NF*^*fx*/−^ SC at 4 mpi (Figures 6K,N,O). By 6 mpi, the frequency and level of axonal degeneration increased in *SLICK-H-CreER;NF*^*fx*/−^ (Figures 6L,P–R). Quantification showed significantly more abnormal axons in *SLICK-H-CreER;NF*^*fx*/−^ mice at 4 mpi (28.8 ± 9.1%) and 6 mpi (48.3 ± 9.5%) in comparison to controls (2.9 ± 0.6%, Figure 6T). Additionally, longitudinal sections were examined where control fibers always maintained a clear nodal gap flanked by paranodal axo-glial loops. In contrast, *SLICK-H-CreER;NF*^*fx*/−^ mice in both the PNS and CNS often had disrupted nodes with myelin inclusions within the axons (*data not shown*). Together, the ultrastructural analyses reveal NF186 is essential for maintaining proper axonal architecture at the nodal region and that destabilization of adult nodes over time contributes to axonal pathology and degeneration.

**Figure 7:**
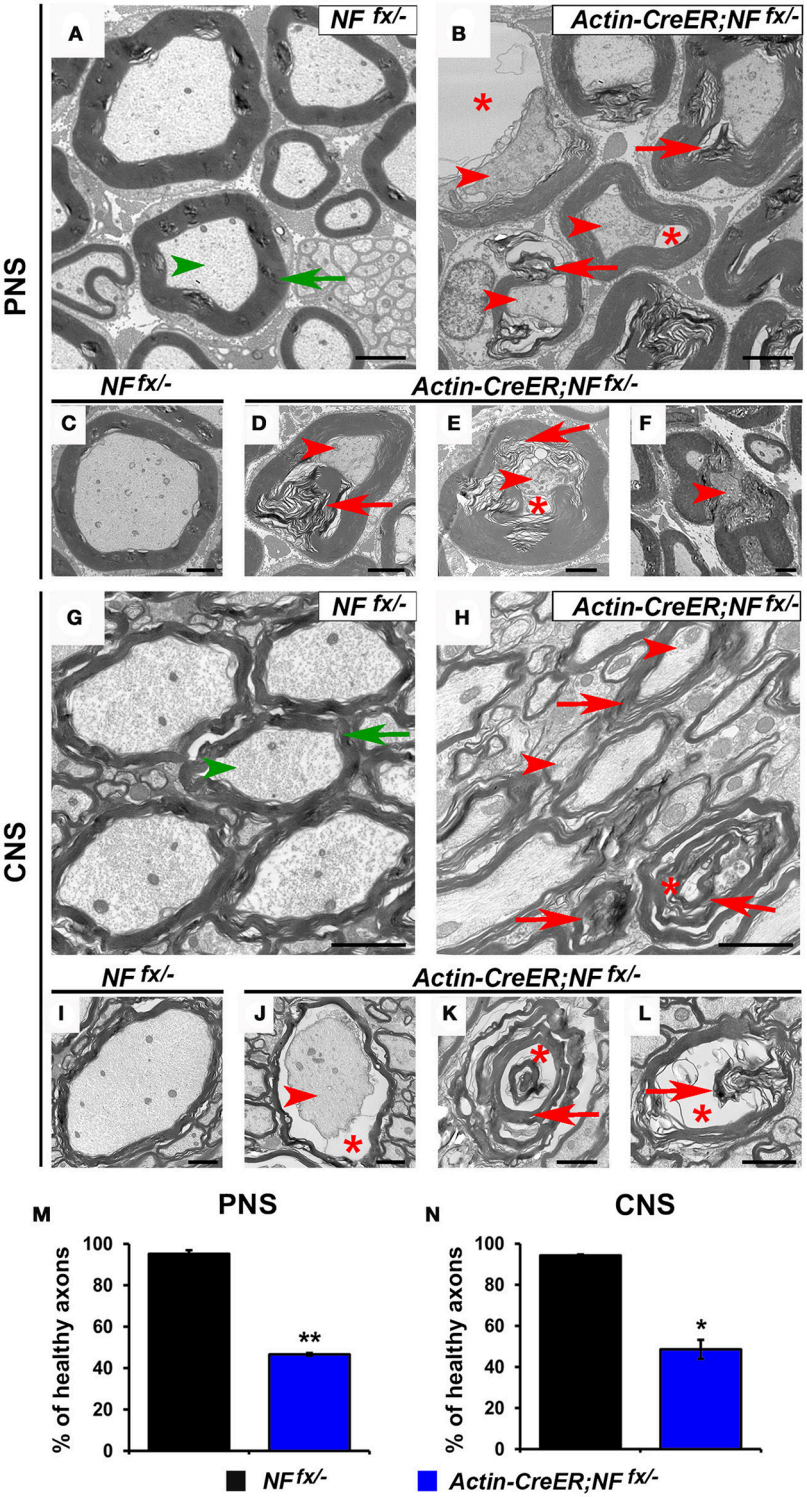
**Destabilization of paranodes along with nodes enhances degeneration of adult myelinated axons. (A–F)** Transmission electron microscopy (TEM) of cross sections from SNs of *NF*^*fx*/−^ control mice **(A,C)** and *Actin-CreER;NF*^*fx*/−^ mice **(B,D–F)** at 2 mpi. Green arrowheads point to normal axons, red arrowheads to axonal pathology, greens arrows to compact myelin, red arrows to abnormal myelin inclusions, and red asterisks to vacuoles within axons. **(G–L)** TEM of cross sections from SCs of *NF*^*fx*/−^ control mice **(G,I)** and *Actin-CreER;NF*^*fx*/−^ mice **(H,J–L)** at 2 mpi. **(M–N)** Quantification of healthy axons found in the cross sections of 2 mpi *NF*^*fx*/−^ (black bar) and *Actin-CreER;NF*^*fx*/−^ (blue bar) SN and SC, respectively. A minimum of 200 axons were scored for SN and 500 axons for SC of each mouse. All data are represented as mean ± SEM (*n* = 3 mice/ group). Scale bar, 2 μm. Black asterisks indicate statistical differences between control and mutant.

As *Actin-CreER*;*NF*^*fx*/−^ mice do not survive past 3 mpi, we processed SNs and SCs of 2 mpi *NF*^*fx*/−^ and *Actin-CreER;NF*^*fx*/−^ for electron microscopy (Figure 7). At this timepoint, we found axons displaying signs of degeneration (red arrowheads) and containing abnormal myelin (red arrows) to be prevalent in *Actin-CreER*;*NF*^*fx*/−^ SN (Figures 7B,D–F); which were not present in SNs from aged-matched *NFfx*^/−^ mice (green arrows for myelin and arrowheads for axons, Figures 7A,C). When normal axons surrounded by tight myelin were scored from control and NF186/NF155 ablated SN's, we found *Actin-CreER*;*NF*^*fx*/−^ (53.4 ± 0.6%) showed significantly more abnormal axons at 2 mpi compared to controls (4.8 ± 1.8%, Figure 7M). Likewise in the SC, control axons appeared normal (Figures 7G,I); however, abnormal and degenerated axons, where only myelin (red arrows) and vacuoles (red asterisks) remained, were frequently observed in *Actin-CreER*;*NF*^*fx*/−^ mice. Quantification found more abnormal axons in *Actin-CreER*;*NF*^*fx*/−^ at 2 mpi (51.3 ± 4.6%) compared to controls (5.8 ± 0.5%, Figure 7N). Of note, although significant axonal disruption was observed when NF186 alone or in combination with NF155 was ablated, it was not accompanied by widespread demyelination. Taken together, these data demonstrate the importance of NF186 and NF155 in the long-term maintenance of the nodal region, and that simultaneous destabilization of the paranodal and nodal complex has dramatic consequences on myelinated axons leading to ultrastructural changes associated with axonal degeneration. These data also provide insight into how changes in axonal domain structure either due to loss of nodal or paranodal proteins can result in the degeneration of myelinated axons.

## Discussion

The node specific cell adhesion molecule, NF186, has emerged as the key player in the initial nodal organization by providing a molecular link between its extracellular partners and intracellular cytoskeletal scaffolding proteins (Thaxton et al., 2011). Other mechanisms have also been suggested in nodal organization that involve contribution of the paranodes along with other nodal proteins (Susuki et al., 2013). In the current study, we have utilized spatiotemporal tools to address the role of neuronal NF186 and paranodal NF155 in the stability of the node, and the timeline in which the nodal complex remains stable after their ablation. Our findings indicate that the long-term nodal maintenance is highly dependent on the stability of NF186 within the nodal complex, and that the paranodal domains *per se* are not required for life-long maintenance of the node. Most importantly, when NF186 is ablated in combination with paranodal NF155, there is accelerated loss of NF186 which coincides with enhanced nodal destabilization, severe reduction in NCV and ultimately degeneration of myelinated axons.

### NF186 stability and requirement in the maintenance of the node

Previous adult ablation studies suggested that NF186 is not required to maintain the nodal complex in either the CNS or PNS (Desmazieres et al., 2014), as other proteins including Na_V_ channels, AnkG, and βIV Spec were retained at the nodes albeit at lower levels 16 weeks after tamoxifen induced ablation (wpi), while NF186 appeared to be absent from the node by 8 wpi. Interesting using the same exact tamoxifen inducible *CreER; Nfasc*^*fx*/−^, it was reported that NF186 was remarkably stable at nodes of Ranvier and NF186 was shown to be present at low levels 16 wpi in nodes of cerebellar white matter tracts (Zonta et al., 2011). Consistent with these observations after tamoxifen-induced ablation, in the *SLICK-H-CreER;NF*^*fx*/−^ it took up to 4 months before NF186 levels began to decline significantly and up to 6 months before NF186 became undetectable at majority of nodes in both PNS and CNS. The resistance of nodal NF186 to degradation after genetic ablation corresponds with the reported 3 month half-life of NF186 after shRNA knockdown from mature nodes in myelinating co-cultures (Zhang et al., 2012). This is in contrast to NF155 stability which was undetectable by 2 mpi from the paranodes in *Plp-CreER;NF*^*fx*/−^ (Pillai et al., 2009) and to NF186 at the cerebellar AIS where it was undetectable in 3 wpi (Zonta et al., 2011). Thus, NF186 integrated into the nodal complex appears to be highly stable with very little turnover.

As NF186 protein levels slowly declined overtime in adults after tamoxifen-induced NF186 ablation, we observed a corresponding reduction in the nodal levels of Na_V_ channels, AnkG, AnkR, and βIV Spec, but not a complete loss of these proteins highlighting the fact that the nodal complex is resistant to destabilization which occurs progressively over months. By this time in animal life, the phenotypes are extremely severe and mutant animals become highly ataxic and immobile, which leads to their death. Similar reductions but not complete loss in Na_V_ channel levels after NF186 ablation from mature nodes were previously reported *in vitro* co-cultures (Zhang et al., 2012) as well as *in vivo* (Desmazieres et al., 2014). Although differential stability of Na_V_ was reported between PNS and CNS in the NF186 adult-ablation model described in Desmazieres et al. (2014) at 4 mpi, our *SLICK-H-CreER;NF*^*fx*/−^ model survived longer and allowed us to further observe nodal destabilization at 6 mpi which was also associated with axonal degeneration. At this later timepoint, no notable differences were observed in the loss of Na_V_ channels or any other nodal protein between the CNS and PNS. This brings into focus whether the differences observed at 4 mpi reflect a differential loss of NF186 in the PNS versus the CNS nodal complex or rather reflect a variance in the accessibility of nodal NF186 to protein degradation between the peripheral and central myelinated axons.

### Nodal stability and contribution of paranodal domains

**Figure 8:**
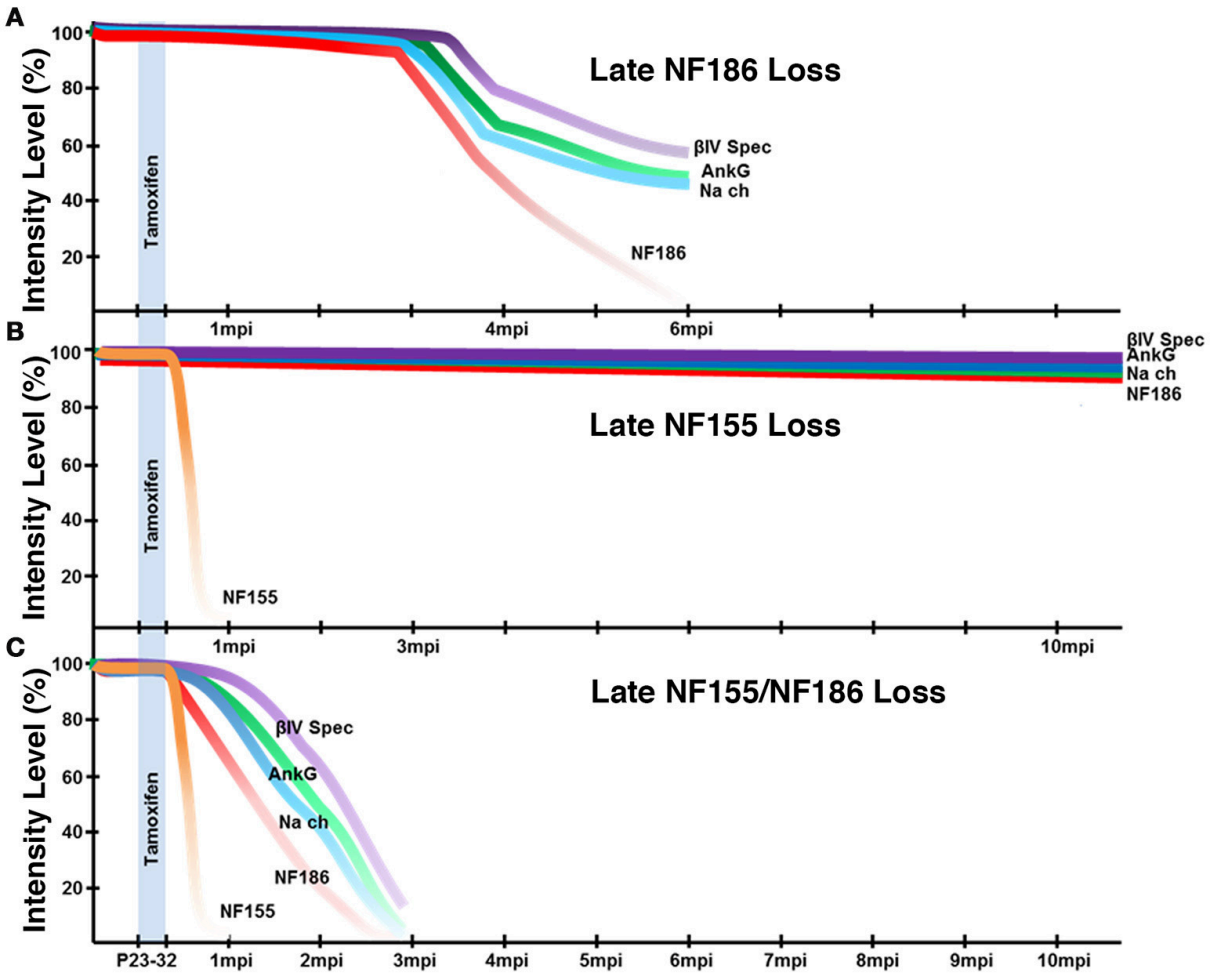
**Schematic representation of nodal destabilization. (A)** Adult ablation of NF186 results in gradual reduction of NF186 which is not completely lost until 6 mpi. As NF186 levels are reduced, nodes fail to maintain normal levels of Na_V_ channels and AnkG followed by βIV Spec. Mice die around 6 mpi. **(B)** Adult ablation of NF155 does not have any appreciable effect on the localization or levels of nodal proteins within 12 mpi. Mice maintain normal lifespan. **(C)** Simultaneous adult ablation of NF155/NF186 results in a rapid loss of NF155 by 1 mpi. Without paranodal NF155, nodes fail to stably maintain NF186 leading to complete loss of NF186 by 3 mpi which is accelerated compared to ablation of NF186 alone. As NF186 levels are reduced, nodes fail to maintain normal levels of Na_V_ channels followed by AnkG and βIV Spec. Mice die around 3 mpi.

Our previous studies showed that loss of paranodes after development did not impact nodal stability (Pillai et al., 2009); however, paranodes were recently proposed to have a distinct role in stabilizing Na_V_ channels at the nodes after NF186 loss (Desmazieres et al., 2014). Loss of paranodes in *Plp-CreER;NF*^*fx*/−^ adults did not have any appreciable effects on the long-term maintenance of the nodal complex even as far as 12 months after ablation indicating that paranodes *per se* are not individually required for nodal stability in either the PNS or CNS (Figure 3). However, the question remained if paranodal axo-glial junctions have a supportive role in maintaining the nodal complex. Our studies reveal loss of paranodes along with NF186 caused accelerated destabilization of the node when compared to loss of NF186 alone in the same timeline (Figure 8). In contrast to the prolonged stability of nodal NF186 after its individual genetic ablation in the *SLICK-H-CreER;NF*^*fx*/−^, NF186 levels dropped more rapidly with the simultaneous ablation of NF186/NF155 in *Actin-CreER;NF*^*fx*/−^ mutants. While at 2 mpi no differences were observed between the intensity of any nodal protein between *NF*^*fx*/−^ and *SLICK-H-CreER;NF*^*fx*/−^ (data not shown); at this time in *Actin-CreER;NF*^*fx*/−^ mutants, significant reduction in the levels of Na_V_ channels, AnkG, and βIV Spec were seen at nodes in both SN and SC. The observation that when trace amounts of NF186 were present at nodal areas, other nodal proteins remained clustered with NF186 at those areas underscores the fact that NF186 is the key stabilizer of the nodal complex. In addition, an increasing number of axons without any detectable nodes or paranodes were observed over time in combined loss of NF186 and NF155. These data demonstrate that without the flanking paranodal domains, the turnover rate of NF186 at the nodes is sharply increased leading to faster nodal destabilization, and further reveal that paranodal axo-glial junctions do not play a determinative role but a supportive role in maintaining life-long stability of the nodal complex in myelinated axons.

While the *Actin-CreER;NF*^*fx*/−^ model with ablation of both neuronal and glial *Nfasc* showed rapid loss of NF186 from nodes and death by 3 mpi; the *SLICK-H-CreER;NF*^*fx*/−^ model with ablation of *Nfasc* only in neurons showed a delayed loss of nodal NF186 and survived until 6 mpi. Although the most probable explanation for the differences between phenotypes and life span of these models is the added loss of paranodal NF155 in the *Actin-CreER;NF*^*fx*/−^, additional variations could have arisen due to genetic backgrounds or other Nfasc functions outside neurons and myelinating glia. Even though both lines were maintained on essentially the same mixed C57BL/6 and 129/Sv strains, the *Actin-CreER;NF*^*fx*/−^ and *SLICK-H-CreER;NF*^*fx*/−^ models were bred and evaluated separately. The only difference that was observed between the two *NF*^*fx*/−^ control groups was the amplitude in *in vivo* tail evoked response at 1 mpi (Figures 5F,L), which is likely explained by the smaller size of the *NF*^*fx*/−^ (*Actin-CreER)* at 1 mpi (Figures 1B, 4B). To account for this and any other variation that could have occurred due to strain variation, the *CreER;NF*^*fx*/−^ were statistically evaluated against their littermate *NF*^*fx*/−^ controls in each experiment. Afterwards, the statistical changes between controls and mutants within each model were compared allowing us to conclude that *Actin-CreER;NF*^*fx*/−^ display enhanced nodal destabilization and quicker demise than the *SLICK-H-CreER;NF*^*fx*/−^ mutants.

### Destabilization of axonal domains and degeneration of myelinated axons

Myelination evolved with axonal domains to promote rapid and energy-efficient conduction of action potentials; however, myelin sheath disruption or loss has unintended consequences on axonal health. As has been observed in several mouse mutants, axonal pathology is often associated with defective axonal domain organization (Dupree et al., 1998; Boiko et al., 2001; Arroyo et al., 2002; Garcia-Fresco et al., 2006). Our studies reported here have uncovered a timeline in which nodal destabilization in adults leads to a significant reduction in nerve impulse propagation, pathology in myelinated axons and ultimately paralysis and subsequent death. *In vivo* NCV assays on peripheral nerves revealed that nodal stability is crucial for proper neuronal activity. Loss of NF186 in adults led to significant alterations in nerve function as conduction velocity was reduced in SN beginning at 4 mpi and worsened by 6 mpi, which is in agreement with reductions in NCV reported at 4 mpi in Desmazieres et al. (2014). In addition, simultaneous loss of the paranodes along with destabilization of the nodal region led to an accelerated decrease in conduction, which highlights the fact that proper nodal maintenance is critical for saltatory nerve conduction by myelinated axons.

The functional relationship between the myelinating cells and the axon also ensures preservation of the axonal ultrastructure and hence axonal function. The presence of ultrastructural abnormalities with degenerating axons in both the PNS and CNS in adult NF186 ablated animals uncovers the importance of the nodal complex in axonal health. Additional support for axonal pathology as a result of axonal domain disorganization comes from the further exacerbated of axonal degeneration in animals with ablation of both NF186 and NF155. Interestingly both models showed the same end stage phenotype, only it was accelerated in the *Actin-CreER;NF*^*fx*/−^ model. Neither model exhibited seizures at any time point or the constant tremor indicative of MBP deficient rodent models (Sidman et al., 1985; Delaney et al., 1995). Instead both mouse models displayed ataxia (only when attempting movement), hindlimb clasping, and abnormal gait which developed into total hind limb paralysis. The mice also exhibited severe kyphosis and muscle atrophy. At end point, the mice were no longer able to right themselves when placed on their backs and appeared to have respiratory distress, although the cause was not investigated. These observations are particularly relevant to human myelin related pathologies where disorganization of established axonal domains serves as a measure of disease progression (Craner et al., 2004; Coman et al., 2006; Desmazieres et al., 2012). Of significant interest are observations that patients with inflammatory demyelinating disease, such as multiple sclerosis, have high levels of antibodies against both NF186 and NF155 (Mathey et al., 2007; Devaux et al., 2012; Ng et al., 2012) raising the possibility that loss of Neurofascin from established axonal domains contributes to degeneration of myelinated axons overtime as is observed in *Actin-CreER;NF*^*fx*/−^ mutants. Since *Actin-CreER;NF*^*fx*/−^ mutants survive an average of 3 mpi, these animals will be valuable to determine whether immunological changes occur that might further trigger demyelination or enhanced disorganization of NF186- and NF155-depedent protein complexes.

Collectively, our findings establish that NF186 functions as the key stabilizer of the nodal complex and that life-long maintenance of functional axonal domains is not only critical for saltatory conduction, but also for preserving the ultrastructure and long-term health of myelinated axons.

## Ethics statement

All animal research was performed with prior approval from UT Health San Antonio's Institutional Animal Care and Use Committee and conforms to the Public Health Service Policy on Humane Care and Use of Laboratory Animals.

## Author contributions

AT: Designed and Performed research and wrote the paper. JS: Performed research. MB: Designed research and wrote the paper.

## Funding

This work was supported by grants from the NIH NIGMS GM063074 (MB), National Multiple Sclerosis Society (MB) and the Zachry Foundation (MB). AT was supported by the NIH NINDS Postdoctoral Fellowship (F32NS092448).

### Conflict of interest statement

The authors declare that the research was conducted in the absence of any commercial or financial relationships that could be construed as a potential conflict of interest.

## Supplementary material

Movie 1 **Loss of NF186 after myelination leads to ataxia and motor coordination defects (A) Video of *SLICK-H-CreER;NF*^*fx*/−^ mouse at 6 mpi**.

Movie 2 **Loss of NF155/NF186 after myelination leads to accelerated ataxia and motor coordination defects (A) Video of *Actin-CreER;NF*^*fx*/−^ mouse at 3 mpi**.
